# Simple Test for Arsenic, Antimony and Phosphorus

**Published:** 1872-01

**Authors:** 


### ARTICLE IX.

### *Simple Test for Arsenic, Antimony and Phosphorus.*

The solution of the substance to be examined is first considerably diluted with water, and poured into a wide-mouthed bottle, to the cork of which are fastened a number of pieces of parchment paper, previously saturated in acetate of lead, nitrate of silver, and sulphate of copper. A few drops of sulphuric acid are now added. some pieces of zinc thrown in, and the cork put in. In case any gases are liberated, they will react upon the strips of paper, and the color will disclose to what particular element the reaction is due. Phosphuretted hydrogen does not blacken nitrate of silver and acetate of lead, but does act upon sulphate of copper· Antimonetted and arsenetted hydrogen do not affect the nitrate of silver and sulphate of copper, but blackens the lead salt. Sulphuretted hydrogen, however, blackens all three of the above metallic solutions. In order to decide what elements are present, the strips of paper are to be macerated in a solution of cyanide of potassium. If the coloration immediately disappears, it was due to the sulphuretted hydrogen; if it slowly changes in cold, and more rapidly in heat, it was caused by phosphorus or antimony; if it only bleaches a little and turns brown, and does not disappear when heated, it may be tracod to arsenic. For ordinary purposes and rapidity of work, this method appears to be sufficiently accurate, and will enable the operator to dispense with the more cumbersome Marsh appartus.—*Med. and Surgical Reporter.*

### MONTHLY SUMMARY.

